# Association of P‐Wave Parameters With Left Atrial Hemodynamics in Atrial Cardiomyopathy

**DOI:** 10.1111/anec.70145

**Published:** 2025-12-18

**Authors:** Melissa Kazantzi, Aljoscha Mohr, Ruth Schneider, Adnan Labedi, Niklas Bach, Johann Rößler, Stephan Salmen, Ralf Gold, Arash Haghikia, Fabienne Kreimer, Michael Gotzmann

**Affiliations:** ^1^ Department of Cardiology and Rhythmology University Hospital St. Josef Hospital Bochum Germany; ^2^ Department of Neurology University Hospital St. Josef Hospital Bochum Germany; ^3^ Department of Cardiology II—Rhythmology University Hospital Münster Germany

**Keywords:** advanced interatrial block, atrial cardiomyopathy, P‐wave parameters

## Abstract

**Background:**

P‐wave parameters, readily obtainable from standard 12‐lead ECGs, have been associated with atrial fibrillation (AF), ischemic stroke, and other cardiovascular conditions. Left atrial cardiomyopathy (AtCM), characterized by atrial fibrosis and functional impairment, is considered a central substrate in the development of AF and embolic stroke of undetermined source. This study examines the relationship between P‐wave parameters and left atrial hemodynamics and evaluates their potential diagnostic utility in identifying AtCM.

**Methods:**

We conducted a monocentric, prospective study in hospitalized patients. Inclusion criteria were sinus rhythm and age ≥ 18 years. P‐wave parameters were assessed in conjunction with echocardiographic measures of left atrial function. Statistical analyses compared patients with and without pathological P‐wave parameters.

**Results:**

A total of 416 patients were included. Pathological P‐wave parameters were highly prevalent, with 55% of patients exhibiting ≥ 3 abnormalities. Advanced interatrial block (IAB) showed a robust association with impaired left atrial hemodynamics, whereas other parameters, such as PTFV1, demonstrated only weak correlations. Patients with advanced IAB exhibited significant alterations in left atrial size, function, and NT‐proBNP levels.

**Conclusions:**

Advanced IAB emerged as the most reliable P‐wave parameter for detecting left atrial dysfunction in AtCM, whereas other P‐wave indices, including PTFV1, were less informative. These findings highlight the diagnostic value of advanced IAB in identifying AtCM, particularly in patients with embolic stroke of undetermined source, and emphasize the need for more refined diagnostic criteria in future investigations.

## Graphical abstract

In the present study, the validity of seven established P‐wave parameters in relation to left atrial hemodynamics was investigated. Our study suggests that advanced IAB is the strongest indicator of altered left atrial hemodynamics and, thus, AtCM.

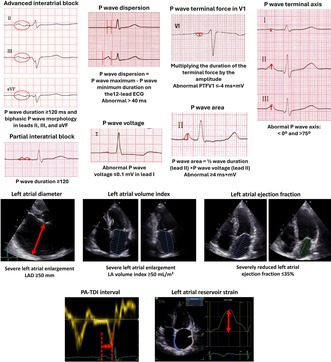

## 1 Introduction

P‐wave parameters can be readily assessed from standard 12‐lead ECGs, making them well suited for investigation in large epidemiological studies (Chen et al. 2022). Their association with the future development of atrial fibrillation (AF) and ischemic stroke is of particular clinical relevance (Kamel et al. 2014, 2015; Nielsen et al. 2015; Maheshwari et al. 2017; Martínez‐Sellés et al. 2020; Marks et al. 2021). In addition, various P‐wave parameters have been linked to left atrial dilatation, AF recurrence after pulmonary vein isolation, and even the incidence of dementia and sudden cardiac death (Chen et al. 2022; Kreimer and Gotzmann 2022).

The underlying condition that may unify these observed associations is left atrial cardiomyopathy (AtCM), a composite entity characterized by atrial fibrosis as well as mechanical and electrical dysfunction (Kreimer and Gotzmann 2022). AtCM is closely associated with AF and ischemic stroke. In 2024, a clinical consensus statement defining AtCM was published by the European Heart Rhythm Association (EHRA) of the ESC, together with the Heart Rhythm Society (HRS), the Asian Pacific Heart Rhythm Society (APHRS), and the Latin American Heart Rhythm Society (LAHRS) (Goette et al. 2024). This document highlights the relevance of P‐wave parameters for diagnosing AtCM, as they reflect left atrial electrical remodeling. However, no single P‐wave parameter has yet been established as a stand‐alone diagnostic tool for AtCM.

In the ARCADIA study, patients with embolic stroke of undetermined source (ESUS) were evaluated for suspected AtCM. One of the criteria applied was a pathological P‐wave terminal force in lead V1 (PTFV1). Although the trial did not demonstrate a benefit of oral anticoagulation, it renewed interest in P‐wave parameters as diagnostic markers for AtCM (Kamel et al. 2024).

Recently, a Consensus Document endorsed by the International Society of Electrocardiology and the International Society for Holter and Noninvasive Electrocardiology summarized the evidence supporting seven established P‐wave parameters (Chen et al. 2022). These indices have been associated with various clinical endpoints in multiple studies (Kamel et al. 2014, 2015; Nielsen et al. 2015; Maheshwari et al. 2017; Martínez‐Sellés et al. 2020; Marks et al. 2021; Kreimer and Gotzmann 2022). Furthermore, several P‐wave parameters correlate with left atrial dilatation and function. These findings support the hypothesis that impaired electrical excitation, in combination with mechanical remodeling of the left atrium, may indicate the presence of AtCM (Goette et al. 2024).

To date, however, no study has systematically evaluated the hemodynamic consequences of the seven described P‐wave parameters. Of particular clinical importance is the question of which parameter most reliably reflects impaired left atrial function. This is especially relevant for future research on AtCM, including studies involving patients with ESUS.

The aim of this study was therefore to investigate the association between abnormal P‐wave parameters, as indicators of electrical dysfunction, and left atrial hemodynamic alterations.

## 2 Methods

In this prospective, monocentric study, consecutive patients were enrolled between July 2022 and November 2024. The present work represents an analysis of our ESUS/AtCM study, conducted at our institution through an interdisciplinary collaboration between cardiologists and neurologists. We examined hospitalized patients across a broad clinical spectrum. Patients without manifest cardiac disease, as well as patients with paroxysmal or persistent AF, ESUS, cardioembolic stroke, or stroke of competing etiologies, were included. All patients provided written informed consent. The study was approved by the local ethics committee (registration number 22‐7535).

### 2.1 Inclusion and Exclusion Criteria

Inclusion criteria for this study were: (1) sinus rhythm at the time of examination and (2) age ≥ 18 years. Exclusion criteria comprised: (1) severe valvular disease, (2) use of antiarrhythmic drugs, (3) previous pulmonary vein isolation (PVI) or cardiac surgery, (4) continuous pacemaker stimulation or any rhythm other than sinus rhythm, (5) electrical or medical cardioversion within the preceding 3 months, (6) end‐stage renal disease, and (7) severely reduced ejection fraction (EF ≤ 30%).

### 2.2 Examinations

The medical history of all patients was recorded, and blood samples were obtained and stored. A 12‐lead ECG was performed immediately before or after the echocardiographic examination.

### 2.3 ECG

The ECGs were recorded at a paper speed of 50 mm/s and an amplitude of 1 mV/cm. All ECGs were analyzed by two independent investigators blinded to the echocardiographic results. In case of disagreement, a third investigator performed a consensus review. P‐wave parameters were assessed according to current recommendations of the International Society of Electrocardiology (Chen et al. 2022). We recorded heart rate, P‐wave duration, PR interval, and QRS duration.

**FIGURE 1:**
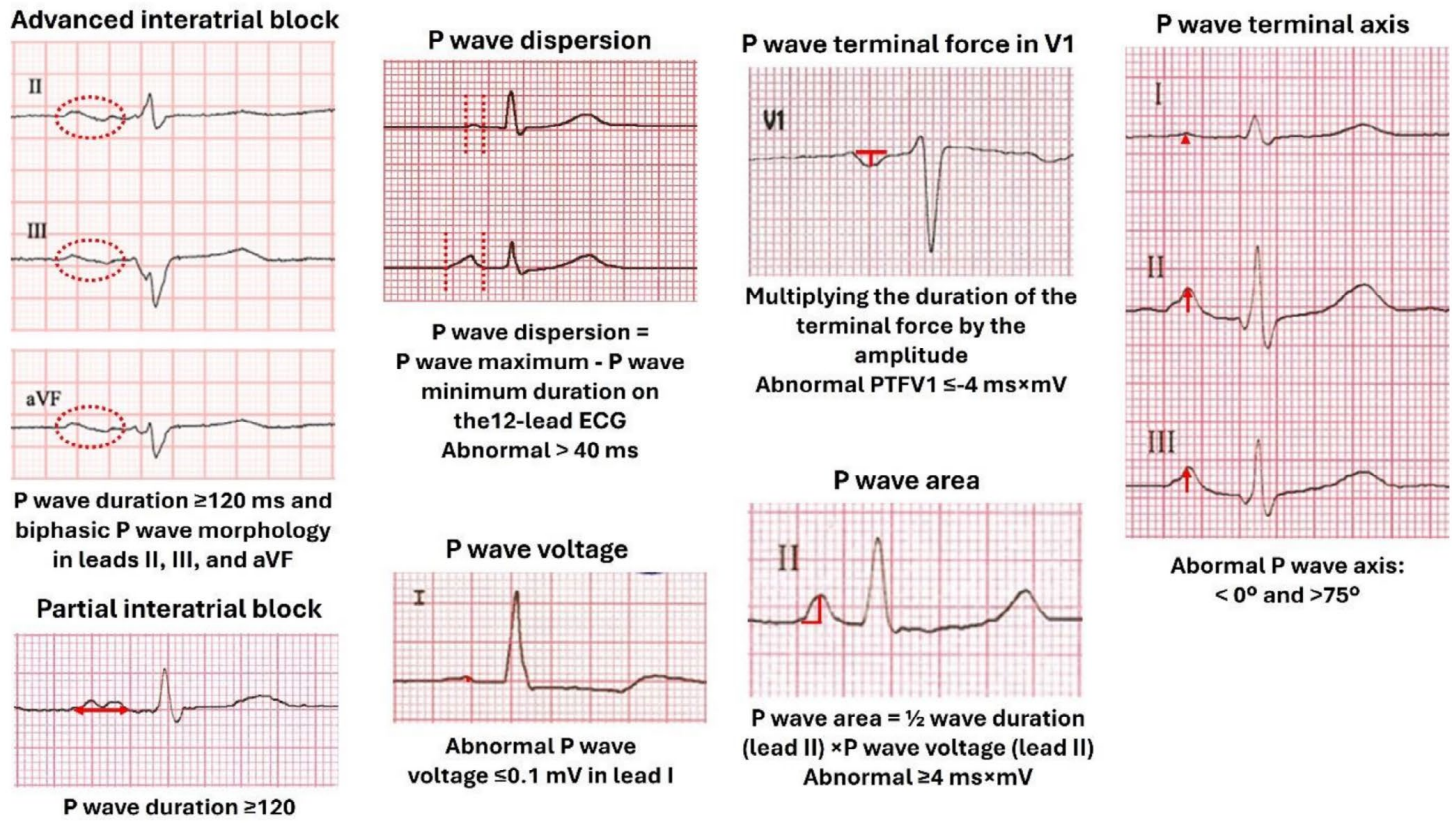
Pathological P‐wave parameters according to the current recommendations of the International Society of Electrocardiography.

The following specific parameters were evaluated:

1. Presence of advanced interatrial block (IAB), defined as a P‐wave duration ≥ 120 ms combined with a biphasic P‐wave in leads II, III, and aVF.
2. Partial IAB, defined as a P‐wave duration ≥ 120 ms.
3. P‐wave dispersion (difference between maximum and minimum P‐wave duration).
4. Abnormal P‐wave in lead I (P‐wave amplitude ≤ 0.1 mV).
5. PTFV1 (duration of the terminal negative portion of the P‐wave in lead V1 multiplied by its depth).
6. P‐wave area (half of the P‐wave duration multiplied by the amplitude in lead II).
7. P‐wave axis (Figure 1).

Patients were categorized into two groups based on the presence or absence of these seven ECG parameters.

### 2.4 Echocardiography

Transthoracic echocardiography was performed by an experienced investigator in accordance with current recommendations of the European Society of Cardiology (Lang et al. 2015; Badano et al. 2018). Examinations were conducted using Vivid E9 or Vivid E95 systems (GE HealthCare, Horten, Norway). Images were archived in a GE Healthcare database and analyzed with the post‐processing software EchoPAC (GE Healthcare). Left atrial diameters were measured in the parasternal long‐axis view. Left atrial and left ventricular volumes were assessed in the apical 4‐ and 2‐chamber views. The PA‐TDI interval was determined using simultaneous ECG registration and the maximum late diastolic excursion of the lateral mitral annulus (Müller et al. 2021). Left atrial strain and its components (conduit strain and contractile strain) were analyzed using EchoPAC in the apical 4‐ and 2‐chamber views (Voigt et al. 2020).

**FIGURE 2:**
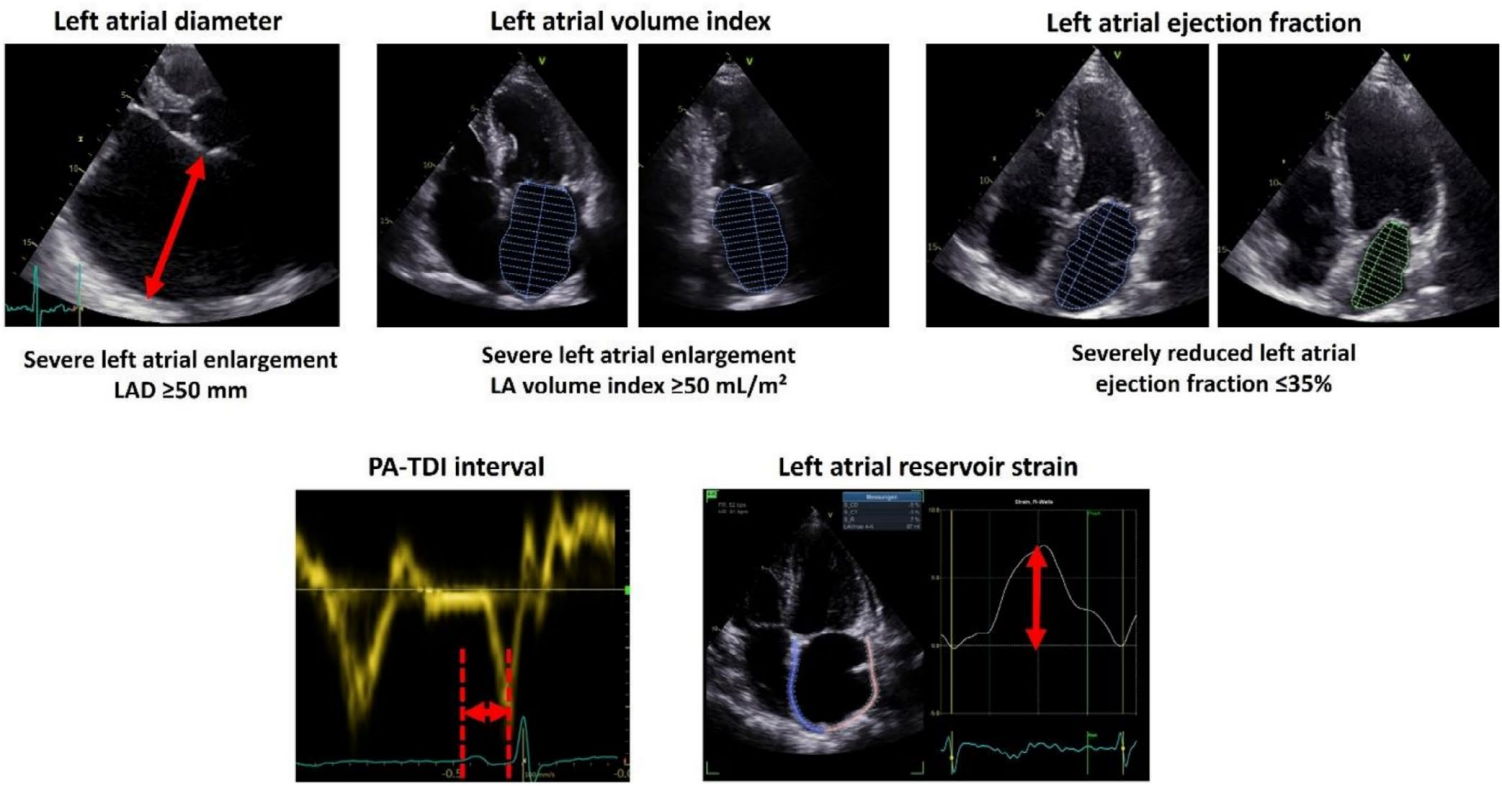
Echocardiographic parameters indicating atrial cardiomyopathy.

In this study, the echocardiographic parameters PA‐TDI, maximum left atrial diameter (LAD), maximum left atrial volume index (LAVI), left atrial ejection fraction (LAEF), and left atrial reservoir strain (LASr) were analyzed (Figure 2). These parameters were selected to assess left atrial function and were examined for significant differences across the various P‐wave parameters.

### 2.5 Statistics

Statistical analyses were performed using SPSS version 29. Numerical data are presented as mean ± standard deviation. Group comparisons of continuous variables were conducted using an unpaired t‐test for normally distributed data and the Mann–Whitney *U* test or Kruskal–Wallis test for non‐normally distributed data. Categorical variables were compared using either *χ* ^2^ analysis or Fisher's exact test. A *p* < 0.05 was considered statistically significant. All reported *p*‐values are two‐sided.

## 3 Results

A total of 416 patients (43.5% women; mean age 69.3 ± 13.7 years) were included in the present study. Of these, 127 patients were enrolled due to ESUS (30.5%), 101 patients due to a history of AF (24.2%), 18 patients because of cardioembolic stroke attributable to AF (4.3%), 26 patients due to micro‐ or macroangiopathic stroke (6.3%), 24 patients with various other conditions (5.8%), and 120 patients without manifest cardiac disease (28.8%). The clinical characteristics of the cohort are summarized in Table 1.

**TABLE 1:** Clinical characteristics of the study cohort (*n* = 416).

Age (years)	69.3 ± 13.7
Women (♀), *n* (%)	181 (43.5)
Body mass index (kg/m^2^)	26.9 ± 5.1
Systolic blood pressure (mmHg)	135 ± 23
Diastolic blood pressure (mmHg)	78 ± 14
Medical history	
Hypertension, *n* (%)	281 (68)
Diabetes mellitus, *n* (%)	86 (21)
Hypercholesterinaemia, *n* (%)	138 (33)
Stroke, *n* (%)	171 (41)
Atrial fibrillation, *n* (%)	119 (29)
Coronary artery disease, *n* (%)	39 (9)
Myocardial infarction, *n* (%)	16 (4)
CHA_2_DS_2_‐VA Score	2.9 ± 1.7
Laboratory	
Creatinine (mg/dL)	0.95 ± 0.25
TSH (mIU/L) (quartiles)	1.29 (0.8, 2)
NT‐pro‐BNP (pg/mL) (quartiles)	212 (83, 559)

Abbreviations: NT‐pro‐BNP, N‐terminal prohormone of B‐natriuretic peptide; PTFV1, P terminal force in V1; TSH, thyroid‐stimulating hormone.

**FIGURE 3:**
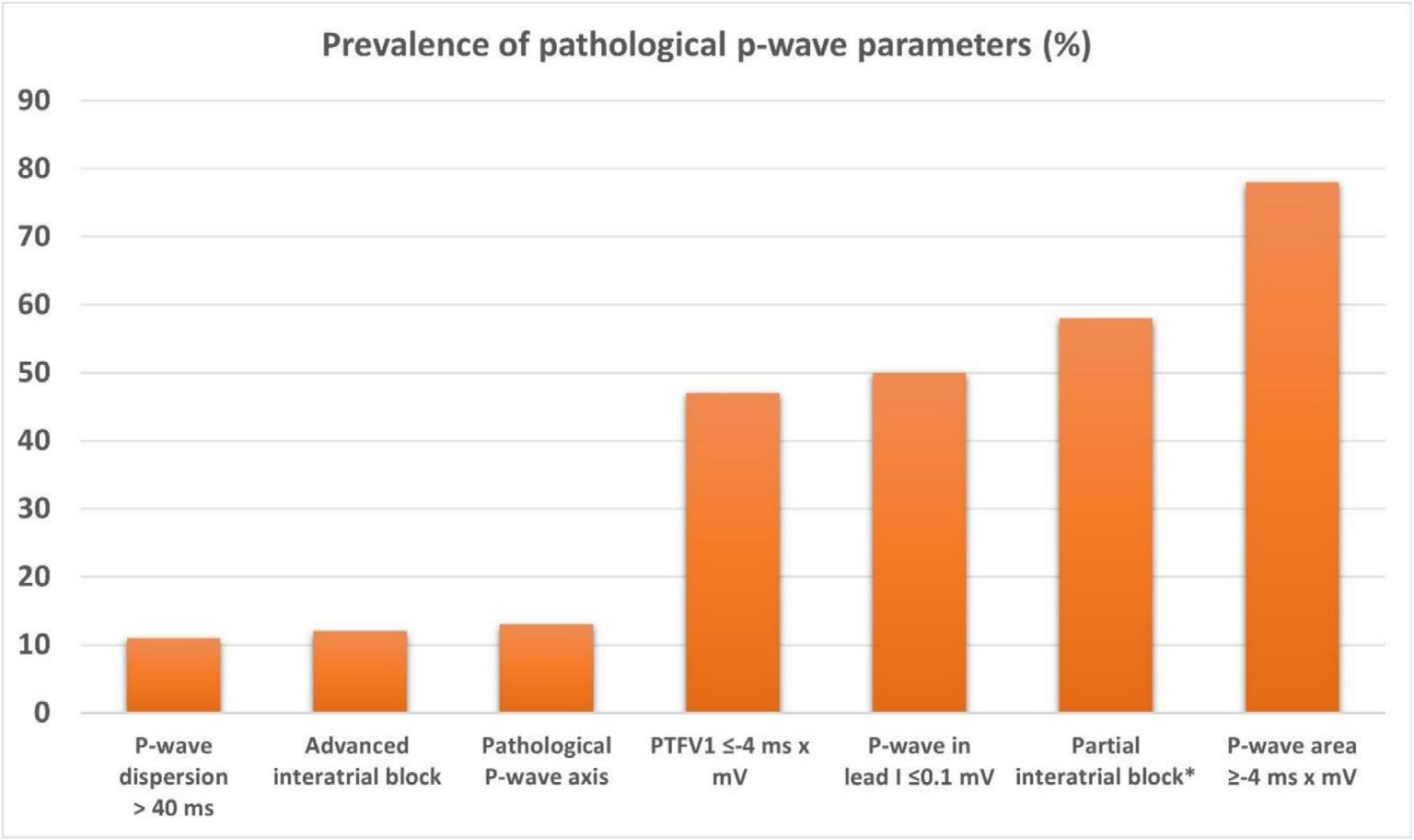
Prevalence of pathological P‐wave parameters in study patients (*n* = 416).

The following pathological P‐wave parameters were observed: advanced IAB, *n* = 50 (12%); partial IAB, *n* = 243 (58%) (patients with advanced IAB included); P‐wave dispersion > 40 ms, *n* = 47 (11%); abnormal P‐wave in lead I ≤ 0.1 mV, *n* = 210 (50%); PTFV1 ≤ −4 ms × mV, *n* = 197 (47%); P‐wave area ≥ −4 ms × mV, *n* = 324 (78%); and pathological P‐wave axis, *n* = 55 (13%) (Figure 3).

Only 10 patients (2.4%) had no pathological P‐wave parameter. Most patients exhibited multiple abnormalities, with ≥ 3 pathological parameters present in approximately 55% of the cohort.

**FIGURE 4:**
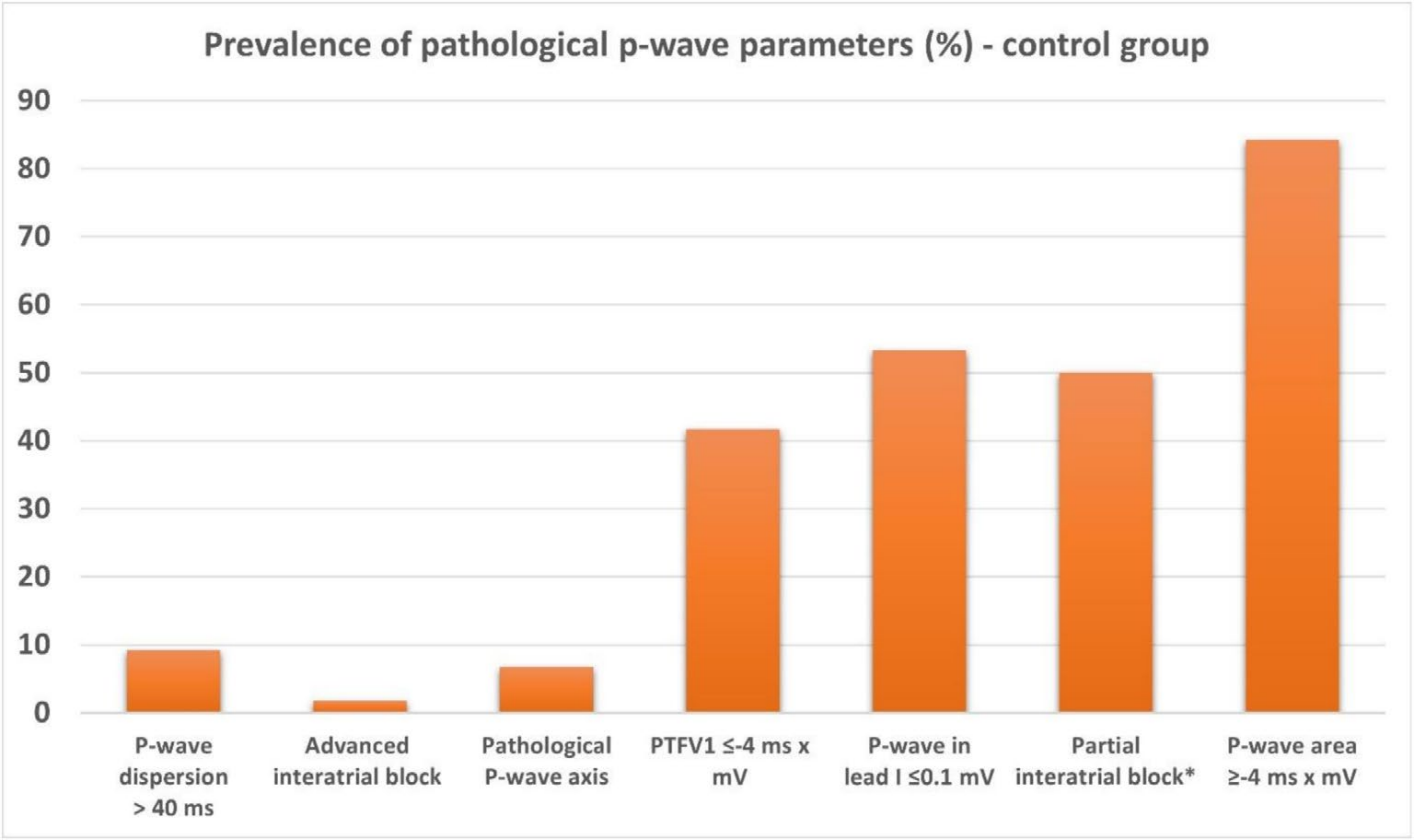
Prevalence of pathological P‐wave parameters in control group (*n* = 120).

In a subgroup analysis, the 120 patients without manifest cardiovascular disease were evaluated. The frequency of pathological P‐wave parameters in this subgroup is shown in Figure 4. Within this control group, only 4 patients (3.3%) demonstrated no pathological P‐wave parameters.

All patients were divided into two groups according to the presence or absence of each of the seven pathological P‐wave parameters (Table 2, S1–S6).

**TABLE 2:** Differentiation of patients based on the ECG parameter advanced interatrial block.

	Advanced interatrial block (*n* = 50)	No advanced interatrial block (*n* = 366)	*p*
Age (years)	77.6 ± 11.2	68.2 ± 13.7	< 0.001
Women (♀), *n* (%)	23 (46)	158 (43)	0.705
Body mass index (kg/m^2^)	27.1 ± 4.6	26.9 ± 5.2	0.813
CHA_2_DS_2_‐VA Score	3.9 ± 1.5	2.8 ± 1.7	< 0.001
NT‐pro‐BNP (pg/mL) (quartiles)	507 (266; 873)	198 (70; 513)	< 0.001
Left ventricular mass index (g/m^2^)	107 ± 27	96 ± 28	0.019
Left ventricular ejection fraction (%)	57.1 ± 7	56.6 ± 6.3	0.596
E/A	1.23 ± 1	0.96 ± 0.51	0.079
E′	8 ± 1.8	9.3 ± 2.8	0.003
A′	9.1 ± 3.1	11.7 ± 3	< 0.001
S′	8.7 ± 1.8	10.2 ± 2.5	< 0.001
E/E′	10.3 ± 4.6	8.3 ± 3.8	0.001
PA‐TDI (ms)	163 ± 28	143 ± 22	< 0.001
LAD maximal (mm)	38.9 ± 6.1	35.4 ± 5.4	< 0.001
LAD minimal (mm)	31.1 ± 6.3	27.4 ± 5.4	< 0.001
LAVI maximal (mL/m^2^)	35.7 ± 12.4	28.3 ± 10.7	< 0.001
LAVI minimal (mL/m^2^)	20.3 ± 11.3	14.2 ± 8	< 0.001
LAEF (%)	45 ± 13	52 ± 12	< 0.001
LASr (%)	17 ± 6.8	22.1 ± 8	< 0.001
LAScd (%)	−9.2 ± 3.8	−10.4 ± 5.8	0.190
LASct (%)	−7.5 ± 7.2	−11.6 ± 5.8	< 0.001

Abbreviations: LAD, left atrial diameter; LAEF, left atrial ejection fraction; LAScd, left atrial conduit strain; LASct, left atrial contractile strain; LASr, left atrial reservoir strain; LAVI, left atrial volume index; NT‐pro‐BNP, N‐terminal prohormone of B‐natriuretic peptide.

**FIGURE 5:**
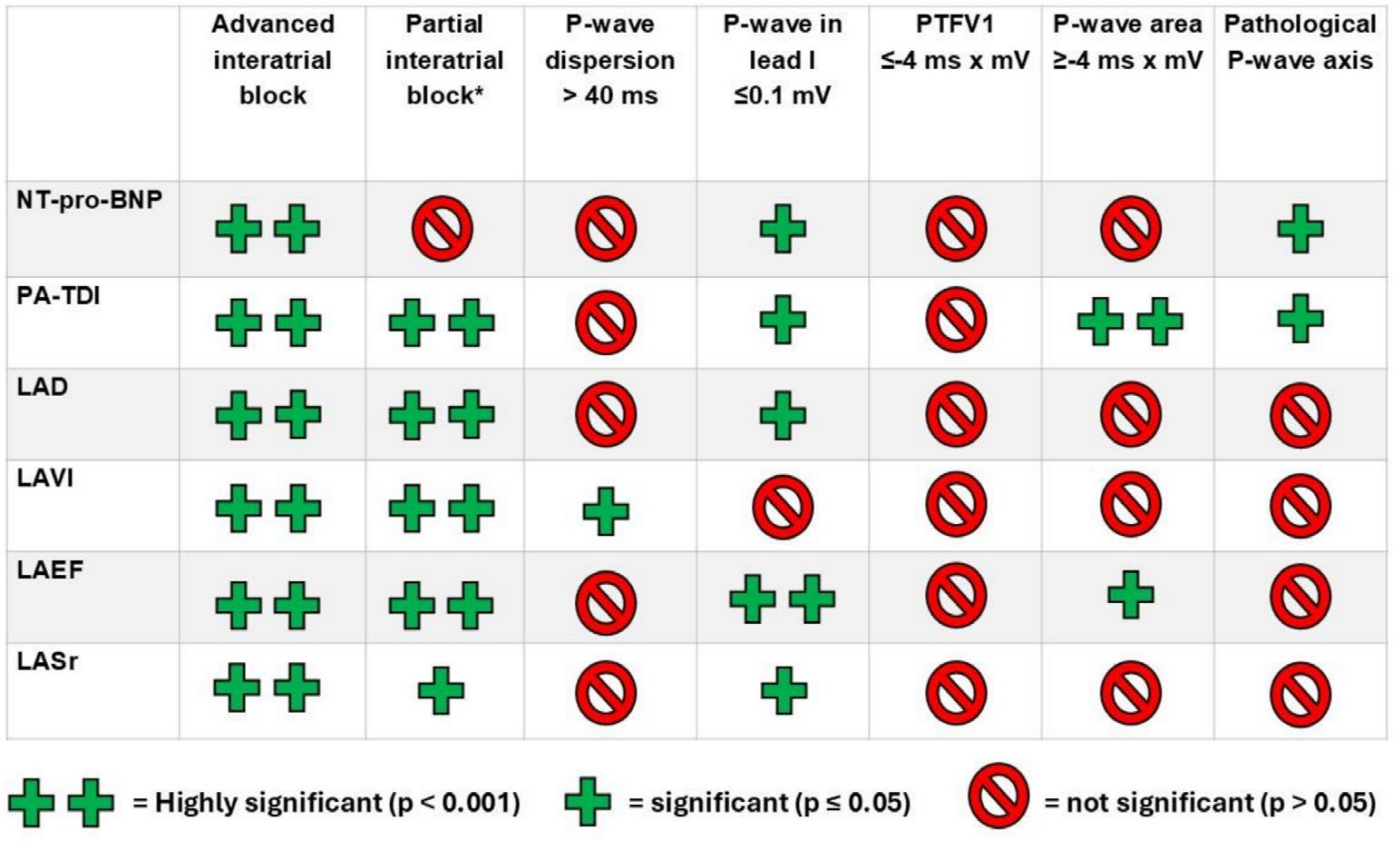
Overview of the associations between pathologic P‐wave parameters and echocardiographic signs of atrial cardiomyopathy.

A summary of the associations between pathological P‐wave parameters, selected echocardiographic markers of left atrial function, and NT‐proBNP levels is presented in Figure 5. Patients with advanced IAB demonstrated highly significant differences across all analyzed parameters (*p* < 0.001). A detailed comparison between patients with and without advanced IAB is provided in Table 2. Patients with partial IAB also showed significant differences compared with those without partial IAB (Table S1); however, this group also includes individuals with advanced IAB.

The remaining P‐wave parameters did not reliably identify patients with impaired left atrial hemodynamics (Figure 5 and Tables S2–S6). In particular, differentiation based on an abnormal PTFV1 (threshold ≤ −4 ms × mV) does not appear to be a dependable marker of left atrial function.

## 4 Discussion

In this prospective study, we systematically investigated the association between seven established P‐wave parameters and left atrial hemodynamics. The main findings were:

1. Pathological P‐wave parameters were highly prevalent among hospitalized patients, with fewer than 5% exhibiting no abnormal P‐wave parameter (Figures 3 and 4).
2. The ECG criterion of advanced interatrial block (IAB) was the most reliable marker for identifying patients with impaired left atrial hemodynamics, whereas other P‐wave parameters showed only partial associations with altered atrial function (Figure 5).
3. Pathological PTFV1 was not a suitable marker for detecting altered left atrial hemodynamics in our study cohort (Table S4).

### 4.1 Atrial Cardiomyopathy and P‐Wave Parameters

AtCM has become an increasing focus of cardiovascular research in recent years (Kreimer and Gotzmann 2022; Goette et al. 2024; Hart et al. 2017) and is associated with AF and stroke. Particularly in patients with ESUS, AtCM is suspected to be present in a relevant subset of cases and may contribute to stroke independently of AF (Hart et al. 2017; Ntaios et al. 2024; Albers et al. 2021; Sajeev et al. 2020). Due to its clinical significance, a consensus statement was recently published by the European Society of Cardiology (Goette et al. 2024), proposing several diagnostic approaches for AtCM. However, no standardized method for identifying clinically manifest AtCM currently exists.

While advanced diagnostic tools such as MRI, invasive electrophysiology, and histology provide valuable insights, they are impractical for large‐scale screening. By contrast, the ECG is simple, widely available, and well suited for identifying patients with suspected AtCM. A recent consensus document on P‐wave parameters (Chen et al. 2022) summarized seven established indices associated with AF, ischemic stroke, and dementia. In our study, we examined the relationship between mechanical and electrical dysfunction as components of AtCM. Impaired left atrial function in AtCM may contribute to the increased risk of ischemic stroke observed in affected individuals (Kreimer and Gotzmann 2022).

Furthermore, the comprehensive review by Bisbal et al. (2020) highlights the clinical relevance of atrial dysfunction as an independent pathological entity. In their JACC Review Topic of the Week, the authors describe “atrial failure” as a condition characterized by structural, electrical, and mechanical abnormalities that contribute to heart failure, arrhythmogenesis, and thromboembolic events (Bisbal et al. 2020). This conceptual framework aligns with our findings, showing that patients with advanced IAB exhibit both electrical and mechanical remodeling—two central components of atrial cardiomyopathy.

Several echocardiographic parameters have historically been used to diagnose AtCM (Kreimer and Gotzmann 2022; Goette et al. 2024). Current recommendations define thresholds for severe AtCM as: left atrial diameter ≥ 5.0 cm, left atrial volume index (LAVI) ≥ 50 mL/m^2^, and left atrial ejection fraction (LAEF) ≤ 35% (Goette et al. 2024). In addition to maximum LA diameter (Healey et al. 2019; Olshansky et al. 2005; Froehlich et al. 2019; Hirota et al. 2021), maximum LAVI (Gupta et al. 2014; Schaaf et al. 2017; Fatema et al. 2009), and LAEF (Goette et al. 2024), frequently used parameters include the PA‐TDI interval (Müller et al. 2021; Leung et al. 2018) and left atrial strain (Leung et al. 2018; Nielsen et al. 2021; Saha et al. 2011; Kawakami et al. 2019). Elevated NT‐proBNP is also considered a marker of AtCM and was used in the ARCADIA study, although it lacks specificity (Kamel et al. 2024; Sieweke et al. 2020).

To determine the most suitable P‐wave parameter for future studies on AtCM, we evaluated echocardiographic parameters including left atrial strain. Threshold values for pathological P‐wave parameters were applied according to the consensus recommendations (Chen et al. 2022). Patients with advanced IAB exhibited significant alterations in left atrial hemodynamics and NT‐proBNP levels (Table 2). Left atrial diameter, volume, LAEF, left atrial reservoir strain, the PA‐TDI interval, and diastolic function parameters (E/E′, E′, A′) were all impaired. In contrast, left ventricular ejection fraction did not differ between patients with and without advanced IAB. As expected, patients with advanced IAB were significantly older.

Approximately 12% of all patients in our study presented with advanced IAB (Figure 3), a markedly higher prevalence than reported in epidemiological studies linking advanced IAB with ischemic stroke. In the study by O'Neal et al. (2016), only 1.8% of over 14,000 participants had advanced IAB, yet they demonstrated a significantly increased stroke risk over a 22‐year follow‐up (Maheshwari et al. 2017). The higher prevalence in our cohort likely reflects the older age and comorbidities of our hospitalized population. In patients without manifest cardiovascular disease, the prevalence of advanced IAB was only 2% (Figure 4). In a prospective registry, patients with structural heart disease aged ≥ 70 years exhibited a prevalence of advanced IAB of nearly 25% and partial IAB in 35% (Martínez‐Sellés et al. 2020). In that cohort, both advanced and partial IAB were associated with AF and stroke. Consistent with these findings, partial or advanced IAB was present in more than half of our study population. Recently, we also demonstrated a high prevalence of advanced IAB in patients with atrial thrombus or spontaneous echo contrast despite sinus rhythm (Kreimer et al. 2023). These findings suggest that impaired left atrial hemodynamics in advanced—and to a lesser extent partial—IAB may promote AF, atrial thrombogenesis, and ischemic stroke. Patients with abnormal P‐waves and altered hemodynamics therefore exhibit two of the three defining components of AtCM: electrical and mechanical remodeling.

Previous epidemiological studies have shown an association between pathological PTFV1 and ischemic stroke (Kamel et al. 2014, 2015). The Cardiovascular Health Study, which prospectively enrolled more than 3000 adults aged ≥ 65 years (Kamel et al. 2015), reported that about 25% of participants had pathological PTFV1. In our study, pathological PTFV1 (≤ −4 ms × mV) was present in nearly half of all patients. However, in contrast to previous findings, pathological PTFV1 was not useful for identifying patients with disturbed left atrial hemodynamics. No significant differences in any hemodynamic parameters were found between patients with and without PTFV1 ≤ −4 ms × mV (Figure 5, Table S4). This discrepancy may be attributable to differences in patient selection. Our cohort included a high proportion of patients with AF and ischemic stroke, potentially obscuring AtCM‐related changes detectable by PTFV1 in healthier populations. This observation may help explain the negative outcome of the ARCADIA trial, which used abnormal PTFV1, increased LA diameter, or elevated NT‐proBNP to define AtCM in patients with ESUS (Kamel et al. 2024).

## 5 Limitations

Compared with large epidemiological studies on P‐wave parameters, our cohort was relatively small. However, to our knowledge, this is the largest study to date to systematically evaluate the association between pathological P‐wave parameters and left atrial hemodynamics. Importantly, we examined hospitalized patients with and without cardiovascular disease; these individuals are typically older and have more comorbidities than participants in population‐based studies. In particular, the high prevalence of stroke in our cohort should be taken into account when interpreting our results. Consequently, the generalizability of our findings to unselected populations remains uncertain. Nevertheless, the patient groups we investigated are clinically relevant, as ECG‐based identification of AtCM may have therapeutic implications in patients with ESUS.

## Author Contributions

**Melissa Kazantzi:** investigation, validation, project administration, data curation; **Aljoscha Mohr:** investigation, validation, project administration, data curation; **Ruth Schneider:** project administration, writing – review and editing; **Adnan Labedi:** investigation, validation, resources; **Niklas Bach:** validation, data curation, project administration; **Johann Rößler:** validation, data curation, project administration; **Stephan Salmen:** resources, validation, investigation; **Ralf Gold:** investigation, writing – review and editing, resources; **Arash Haghikia:** writing – review and editing, resources, validation; **Fabienne Kreimer:** supervision, writing – original draft; **Michael Gotzmann:** conceptualization, funding acquisition, writing – original draft, methodology, software, formal analysis, project administration, supervision, visualization.

## Conflicts of Interest

The authors declare no conflicts of interest.

## Supporting information


**Appendix S1:** anec70145‐sup‐0001‐AppendixS1.docx.

## Data Availability

The data that support the findings of this study are available on request from the corresponding author. The data are not publicly available due to privacy or ethical restrictions.
