# Supplementary material for: Association of P‐Wave Parameters With Left Atrial Hemodynamics in Atrial Cardiomyopathy
Source: Ann Noninvasive Electrocardiol. 2025 Dec 18;31(1):e70145. doi: 10.1111/anec.70145 (PMC12715353; doi:10.1111/anec.70145)
Supplement: Supplementary file 1 — Appendix S1: anec70145‐sup‐0001‐AppendixS1.docx. [file ANEC-31-e70145-s001.docx]

**Table Supplement 1:** Differentiation of patients based on the ECG parameter partial interatrial block*

|  | Partial interatrial block (n = 243)* | No partial interatrial block  (n = 173) | p value |
| --- | --- | --- | --- |
| Age (years) | 71.3 ± 12.1 | 66.6 ± 15.3 | <0.001 |
| Women (♀), n (%) | 85 (35) | 96 (55) | <0.001 |
| Body mass index (kg/m²) | 27.2 ± 5.3 | 26.6 ± 4.8 | 0.267 |
| CHA_2_DS_2_-VA Score | 3 ± 1.7 | 2.7 ± 1.8 | 0.076 |
| NT-pro-BNP (pg/ml) (Quartiles) | 199 (80; 589) | 238 (82; 556) | 0.689 |
| Left ventricular mass index (g/m²) | 102 ± 31 | 91 ± 21 | <0.001 |
| Left ventricular ejection fraction (%) | 56.8 ± 7 | 56.4 ± 5.3 | 0.575 |
| E/A | 1.01 ± 0.64 | 0.96 ± 0.52 | 0.391 |
| E´ | 8.79 ± 2.21 | 9.71 ± 3.24 | <0.001 |
| A´ | 11.3 ± 3.3 | 11.5 ± 2.7 | 0.709 |
| S´ | 9.89 ± 2.36 | 10.2 ± 2.57 | 0.179 |
| E/E´ | 8.75 ± 3.94 | 8.2 ± 3.96 | 0.164 |
| PA-TDI (ms) | 149 ± 28 | 141 ± 21 | <0.001 |
| LAD maximal (mm) | 36.7 ± 5.7 | 34.4 ± 5.1 | <0.001 |
| LAD minimal (mm) | 28.8 ± 6 | 26.4 ± 9 | <0.001 |
| LAVI maximal (ml/m²) | 30.8 ± 12.2 | 26.7 ± 9 | <0.001 |
| LAVI minimal (ml/m²) | 16.3 ± 9.6 | 12.9 ± 6.6 | <0.001 |
| LAEF (%) | 49.3 ± 12.4 | 53.4 ± 11.1 | <0.001 |
| LASr (%) | 20.5 ± 7.8 | 23.1 ± 8.2 | 0.001 |
| LAScd (%) | -9.3 ± 4.9 | -11.5 ± 6.2 | <0.001 |
| LASct (%) | -11 ± 6.4 | -11.4 ± 5.7 | 0.577 |

* patients with advanced interatrial block (n = 50) were included in this group

NT-pro-BNP, N-terminal prohormone of B-natriuretic peptide; LAD, left atrial diameter; LAVI, left atrial volume index; LAEF, left atrial ejection fraction; LASr, left atrial reservoir strain; LAScd, left atrial conduit strain; LASct, left atrial contractile strain

**Table Supplement 2:** Differentiation of patients based on the ECG parameter pathological P-wave dispersion (> 40 ms)

|  | Pathological P-wave dispersion  (n = 47) | No pathological P-wave dispersion  (n = 369) | p value |
| --- | --- | --- | --- |
| Age (years) | 68.7 ± 15.6 | 69.4 ± 13.5 | 0.740 |
| Women (♀), n (%) | 17 (36) | 164 (44) | 0.281 |
| Body mass index (kg/m²) | 27.2 ± 4.8 | 26.9 ± 5.1 | 0.699 |
| CHA_2_DS_2_-VA Score | 2.9 ± 1.5 | 2.9 ± 1.8 | 0.958 |
| NT-pro-BNP (pg/ml) (Quartiles) | 142 (51; 289) | 299 (86; 577) | 0.072 |
| Left ventricular mass index (g/m²) | 98 ± 28 | 97 ± 28 | 0.818 |
| Left ventricular ejection fraction (%) | 57.9 ± 5 | 56.5 ± 6.5 | 0.146 |
| E/A | 1.07 ± 0.5 | 0.98 ± 0.6 | 0.352 |
| E´ | 9.76 ± 3.32 | 9.1 ± 2.63 | 0.120 |
| A´ | 11.2 ± 3.6 | 11.4 ± 3 | 0.741 |
| S´ | 10.8 ± 2.7 | 9.9 ± 2.4 | 0.036 |
| E/E´ | 8.63 ± 4.53 | 8.5 ± 3.88 | 0.834 |
| PA-TDI (ms) | 149 ± 28 | 145 ± 23 | 0.302 |
| LAD maximal (mm) | 36.9 ± 5.6 | 35.6 ± 5.6 | 0.152 |
| LAD minimal (mm) | 29.2 ± 5.3 | 27.6 ± 5.7 | 0.068 |
| LAVI maximal (ml/m²) | 32.6 ± 13 | 28.7 ± 10.8 | 0.014 |
| LAVI minimal (ml/m²) | 17.8 ± 11.3 | 14.5 ± 8.2 | 0.017 |
| LAEF (%) | 47.7 ± 12.8 | 51.5 ± 11.9 | 0.052 |
| LASr (%) | 20.9 ± 8.5 | 21.7 ± 8 | 0.535 |
| LAScd (%) | -11 ± 6.5 | -10.2 ± 5.5 | 0.356 |
| LASct (%) | -10.5 ± 5.1 | -11.3 ± 6.2 | 0.430 |

NT-pro-BNP, N-terminal prohormone of B-natriuretic peptide; LAD, left atrial diameter; LAVI, left atrial volume index; LAEF, left atrial ejection fraction; LASr, left atrial reservoir strain; LAScd, left atrial conduit strain; LASct, left atrial contractile strain

**Table Supplement 3:** Differentiation of patients based on the ECG parameter abnormal P-wave in lead I (≤0.1 mV)

|  | Abnormal  P-wave in lead I  (n = 210) | No abnormal  P-wave in lead I  (n = 154) | p value |
| --- | --- | --- | --- |
| Age (years) | 71.9 ± 13.3 | 66 ± 13.8 | <0.001 |
| Women (♀), n (%) | 101 (48) | 62 (40) | 0.137 |
| Body mass index (kg/m²) | 26.7 ± 5.2 | 26.9 ± 4.9 | 0.664 |
| CHA_2_DS_2_-VA Score | 3 ± 1.7 | 2.4 ± 1.8 | <0.001 |
| NT-pro-BNP (pg/ml) (Quartiles) | 225 (92; 617) | 182 (54; 448) | 0.048 |
| Left ventricular mass index (g/m²) | 98 ± 26 | 95 ± 30 | 0.269 |
| Left ventricular ejection fraction (%) | 57 ± 6.3 | 56.3 ± 5.8 | 0.286 |
| E/A | 1 ± 0.54 | 0.95 ± 0.5 | 0.305 |
| E´ | 9.23 ± 2.8 | 9.12 ± 2.8 | 0.705 |
| A´ | 11 ± 3.21 | 12 ± 2.83 | 0.002 |
| S´ | 9.89 ± 2.47 | 10.4 ± 2.44 | 0.075 |
| E/E´ | 8.9 ± 4.29 | 8.17 ± 3.67 | 0.089 |
| PA-TDI (ms) | 148 ± 25 | 142 ± 20 | 0.012 |
| LAD maximal (mm) | 35.9 ± 5.3 | 35.1 ± 5.8 | 0.019 |
| LAD minimal (mm) | 28.2 ± 5.3 | 26.8 ± 5.5 | 0.012 |
| LAVI maximal (ml/m²) | 29.8 ± 11 | 28 ± 11 | 0.139 |
| LAVI minimal (ml/m²) | 15.8 ± 9.3 | 13.4 ± 7.4 | 0.012 |
| LAEF (%) | 49.3 ± 12.4 | 53.6 ± 11.1 | <0.001 |
| LASr (%) | 20.7 ± 7.8 | 23.2 ± 7.9 | 0.003 |
| LAScd (%) | -10 ± 5.6 | -10.8 ± 5.9 | 0.188 |
| LASct (%) | -10.5 ± 5.9 | -12.4 ± 6 | 0.003 |

NT-pro-BNP, N-terminal prohormone of B-natriuretic peptide; LAD, left atrial diameter; LAVI, left atrial volume index; LAEF, left atrial ejection fraction; LASr, left atrial reservoir strain; LAScd, left atrial conduit strain; LASct, left atrial contractile strain

**Table Supplement 4:** Differentiation of patients based on the ECG parameter pathological PTFV1 (≤-4 ms x mV)

|  | Pathological PTFV1 (≤-4 ms x mV)  (n = 197) | No pathological PTFV1  (>-4 ms x mV)  (n = 215) | p value |
| --- | --- | --- | --- |
| Age (years) | 67.7 ± 12.9 | 70.7 ± 14.3 | 0.030 |
| Women (♀), n (%) | 91 (46) | 90 (42) | 0.376 |
| Body mass index (kg/m²) | 26.7 ± 5 | 27.2 ± 5.2 | 0.364 |
| CHA_2_DS_2_-VA Score | 2.8 ± 1.7 | 3 ± 1.8 | 0.368 |
| NT-pro-BNP (pg/ml) (Quartiles) | 210 (77; 526) | 214 (85; 598) | 0.643 |
| Left ventricular mass index (g/m²) | 98 ± 30 | 97 ± 25 | 0.624 |
| Left ventricular ejection fraction (%) | 56.7 ± 6.3 | 56.6 ± 6.5 | 0.888 |
| E/A | 0.95 ± 0.5 | 1.01 ± 0.65 | 0.283 |
| E´ | 9.09 ± 2.66 | 9.29 ± 2.8 | 0.370 |
| A´ | 11.4 ± 3.1 | 11.4 ± 3.1 | 0.824 |
| S´ | 9.91 ± 2.42 | 10.1 ± 2.48 | 0.335 |
| E/E´ | 8.62 ± 3.88 | 8.38 ± 3.97 | 0.534 |
| PA-TDI (ms) | 144 ± 24 | 147 ± 23 | 0.243 |
| LAD maximal (mm) | 35.5 ± 5.7 | 36 ± 5.5 | 0.352 |
| LAD minimal (mm) | 27.7 ± 5.7 | 27.9 ± 5.6 | 0.659 |
| LAVI maximal (ml/m²) | 29.5 ± 12 | 28.8 ± 10.3 | 0.494 |
| LAVI minimal (ml/m²) | 15.4 ± 9.2 | 14.4 ± 8.1 | 0.276 |
| LAEF (%) | 50.4 ± 12.4 | 51.6 ± 11.6 | 0.336 |
| LASr (%) | 21 ± 8.5 | 22.1 ± 7.7 | 0.179 |
| LAScd (%) | -9.8 ± 5.2 | -10.6 ± 5.9 | 0.154 |
| LASct (%) | -11.1 ± 6.4 | -11.3 ± 5.9 | 0.795 |

PTFV1, P terminal force in V1; NT-pro-BNP, NT-pro-BNP, N-terminal prohormone of B-natriuretic peptide; LAD, left atrial diameter; LAVI, left atrial volume index; LAEF, left atrial ejection fraction; LASr, left atrial reservoir strain; LAScd, left atrial conduit strain; LASct, left atrial contractile strain

**Table Supplement 5:** Differentiation of patients based on the ECG parameter pathological P-wave area (≥-4 ms x mV)

|  | Pathological P-wave area (≥4 ms x mV)  (n = 324) | No pathological P-wave area  (≥4 ms x mV)  (n = 92) | p value |
| --- | --- | --- | --- |
| Age (years) | 68.3 ± 13.5 | 72.8 ± 14.1 | 0.005 |
| Women (♀), n (%) | 137 (42) | 44 (48) | 0.344 |
| Body mass index (kg/m²) | 26.7 ± 4.8 | 27.8 ± 6 | 0.061 |
| CHA_2_DS_2_-VA Score | 2.7 ± 1.7 | 3.7 ± 1.8 | <0.001 |
| NT-pro-BNP (pg/ml) (Quartiles) | 207 (80; 540) | 249 (88; 714) | 0.207 |
| Left ventricular mass index (g/m²) | 96 ± 27 | 102 ± 28 | 0.076 |
| Left ventricular ejection fraction (%) | 56.8 ± 6.2 | 56 ± 7 | 0.314 |
| E/A | 0.98 ± 0.52 | 1.03 ± 0.82 | 0.504 |
| E´ | 9.28 ± 2.8 | 8.78 ± 2.4 | 0.131 |
| A´ | 11.5 ± 3 | 11.1 ± 3.2 | 0.273 |
| S´ | 10.1 ± 2.4 | 9.9 ± 2.7 | 0.623 |
| E/E´ | 8.53 ± 3.96 | 8.48 ± 3.96 | 0.912 |
| PA-TDI (ms) | 143 ± 23 | 154 ± 23 | <0.001 |
| LAD maximal (mm) | 35.6 ± 5.5 | 36.2 ± 5.9 | 0.430 |
| LAD minimal (mm) | 27.6 ± 5.6 | 28.5 ± 5.9 | 0.176 |
| LAVI maximal (ml/m²) | 28.6 ± 11.1 | 31.1 ± 11 | 0.064 |
| LAVI minimal (ml/m²) | 14.3 ± 8 | 16.7 ± 10.3 | 0.027 |
| LAEF (%) | 51.7 ± 11.1 | 48.8 ± 14.6 | 0.047 |
| LASr (%) | 21.9 ± 7.6 | 20.4 ± 9.3 | 0.129 |
| LAScd (%) | -10.5 ± 5.6 | -9.6 ± 5.7 | 0.181 |
| LASct (%) | -11.3 ± 6 | -10.8 ± 6.4 | 0.538 |

NT-pro-BNP, N-terminal prohormone of B-natriuretic peptide; LAD, left atrial diameter; LAVI, left atrial volume index; LAEF, left atrial ejection fraction; LASr, left atrial reservoir strain; LAScd, left atrial conduit strain; LASct, left atrial contractile strain

**Table Supplement 6:** Differentiation of patients based on the ECG parameter pathological P-wave axis

|  | Pathological  P-wave axis  (n = 55) | No pathological  P-wave axis  (n = 347) | p value |
| --- | --- | --- | --- |
| Age (years) | 70.2 ± 15.3 | 68.8 ± 13.5 | 0.469 |
| Women (♀), n (%) | 22 (40) | 154 (44) | 0.543 |
| Body mass index (kg/m²) | 25.7 ± 6.1 | 27.1 ± 4.9 | 0.075 |
| CHA_2_DS_2_-VA Score | 3.2 ± 1.5 | 2.8 ± 1.8 | 0.106 |
| NT-pro-BNP (pg/ml) (Quartiles) | 346 (208; 742) | 195 (70; 522) | 0.007 |
| Left ventricular mass index (g/m²) | 101 ± 29 | 97 ± 28 | 0.345 |
| Left ventricular ejection fraction (%) | 56.9 ± 7.7 | 56.7 ± 6.2 | 0.369 |
| E/A | 1.15 ± 0.88 | 0.97 ± 0.54 | 0.037 |
| E´ | 9.06 ± 2.52 | 9.25 ± 2.79 | 0.643 |
| A´ | 10.7 ± 3.7 | 11.5 ± 3 | 0.054 |
| S´ | 9.91 ± 2.89 | 10.1 ± 2.4 | 0.638 |
| E/E´ | 9.14 ± 3.92 | 8.39 ± 4 | 0.204 |
| PA-TDI (ms) | 151 ± 28 | 144 ± 22 | 0.048 |
| LAD maximal (mm) | 36 ± 6 | 35.6 ± 5.4 | 0.627 |
| LAD minimal (mm) | 28.5 ± 6.2 | 27.5 ± 5.5 | 0.219 |
| LAVI maximal (ml/m²) | 27.6 ± 9.1 | 29.1 ± 11.5 | 0.330 |
| LAVI minimal (ml/m²) | 14 ± 7.2 | 14.9 ± 8.8 | 0.479 |
| LAEF (%) | 50.9 ± 13.6 | 51.2 ± 11.8 | 0.870 |
| LASr (%) | 21.7 ± 8 | 21.7 ± 8 | 1.000 |
| LAScd (%) | -11.5 ± 5.6 | -10.1 ± 5.6 | 0.087 |
| LASct (%) | -10.1 ± 6.2 | -11.5 ± 6 | 0.136 |

NT-pro-BNP, N-terminal prohormone of B-natriuretic peptide; LAD, left atrial diameter; LAVI, left atrial volume index; LAEF, left atrial ejection fraction; LASr, left atrial reservoir strain; LAScd, left atrial conduit strain; LASct, left atrial contractile strain
